# Bioresponsive Hyaluronic Acid‐Based Hydrogel Inhibits Matrix Metalloproteinase‐2 in Glioblastoma Microenvironment

**DOI:** 10.1002/cmdc.202401040

**Published:** 2025-06-24

**Authors:** Federica Barbugian, Domenico Salerno, Elisa Ballarini, Luca Crippa, Oscar Francesconi, Francesco Mantegazza, Guido Cavaletti, Stefano Roelens, Gemma Leone, Simone Pepi, Luigi Talarico, Agnese Magnani, Cristina Nativi, Laura Russo

**Affiliations:** ^1^ School of Medicine and Surgery Università di Milano‐Bicocca 20900 Monza (MB) Italy; ^2^ Department of Chemistry University of Florence, Polo Scientifico e Tecnologico Firenze 50019 Sesto Fiorentino Italy; ^3^ INSTM Consorzio Interuniversitario per la Scienza e Tecnologia dei Materiali Via Giusti, 9 50021 Firenze Italy; ^4^ Department of Biotechnology, Chemistry and Pharmacy Università degli Studi di Siena 53100 Siena Italy

**Keywords:** bioinstructive hydrogel, glioblastoma multiforme, hyaluronic acid, microenvironment, MMP‐2, MMP‐9

## Abstract

Glioblastoma multiforme (GBM) is an extremely malignant cancer, resistant to standard therapies. Tumor resection is associated with better outcomes but a complete resection of GBMs is challenging for anatomical limitation and for the high degree of invasiveness. A bioresponsive hydrogel based on hyaluronic acid (HA) cross‐linked with a branched metalloproteinase (MMP) inhibitor (MMPI) is proposed. The fully characterized hydrogel, HA‐MMPI, presents physical properties suitable for filling the surgical resection cavity and for in situ delivery of the inhibitor. The bioresponsive material selectively inhibits MMP‐2 versus MMP‐9 in glioblastoma microenvironment.

## Graphical abstract

A bioresponsive hydrogel based on hyaluronic acid crosslinked with a MMP inhibitor (HA‐MMPI) is generated to modulate the glioblastoma multiforme (GBM) microenvironment. In preliminary tests with 3D U87 models, HA‐MMPI selectively inhibits MMP‐2, reducing cell migration. The design features and preliminary results position the HA‐MMPI hydrogel as a promising candidate for further evaluation in the development of fillers for GBM therapies.

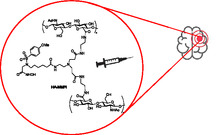

## 1 Introduction

Glioblastoma multiforme (GBM) is the most frequent lethal brain tumor with an overall survival of less than 2 years.^[^ ^1^, ^2^, ^3^ ^]^ GBM onset and progression is generally asymptomatic, making an early diagnosis an urgent medical need.^[^ ^4^ ^]^ Unfortunately, despite the extensive research on the pathophysiology of GBM and the identification and characterization of new therapeutic targets, the prognosis for GBM patients remains poor.^[^ ^5^, ^6^ ^]^

The high neoplastic cells heterogenicity characterizing GBM has recently been reported to contribute to downregulate patients’ immune system and modify the extracellular matrix (ECM) to favor tumor growth and progression.^[^ ^7^ ^]^ Among the ECM components, metalloproteinases (MMPs) are actively involved in GBM invasiveness, mediating the induction of metastasis in distant brain areas.^[^ ^8^, ^9^ ^]^ In particular, MMP‐2 (also known as gelatinase A) is overexpressed in aggressive GBM and increases tumor invasiveness and malignancy by degrading the ECM and promoting cell spreading and vessel maturation.^[^ ^10^, ^11^ ^]^ The level of MMP‐2 has been associated with poor prognosis in patients affected by GBM. The selective inhibition of MMP‐2 is therefore markedly important for the treatment of GBM since, after surgical resection, residual senescence cells can invade the surrounding tissue and reconstitute the tumor microenvironment (TME).^[^ ^12^, ^13^, ^14^ ^]^ However, a systemic administration of MMP inhibitors (MMPIs) is not feasible, because of their intrinsic lack of selectivity and the associated side effects.^[^ ^15^, ^16^, ^17^ ^]^ An effective solution consists in filling the surgical sites, after tumor resection, with a biomaterial progressively releasing in situ the appropriate MMPI.^[^ ^18^ ^]^

Hyaluronic acid (HA) is widely employed as a biomaterial for therapeutic applications.^[^ ^19^, ^20^ ^]^ In this context, HA is one of the main components of the brain ECM, and its parental receptors are overexpressed in GBM cells.^[^ ^21^ ^]^ In recent studies, HA‐based hydrogels were employed to deliver anticancer and immunotherapeutic agents to GBM cells, taking advantage of the HA ability to target specific cells within TME.^[^ ^22^, ^23^ ^]^ In addition, HA hydrogels have been shown to reduce inflammation and promote tissue regeneration,^[^ ^24^, ^25^ ^]^ properties which may greatly help to improve GBM patients’ outcomes.

We recently reported on a bioactive HA functionalized with a water‐soluble MMPI, characterized by: 1) an increased resistance to hyaluronidase degradation, 2) MMP inhibition properties, and3) the ability to prevent dehydration of corneal epithelial cells in vivo.^[^ ^26^ ^]^ Moving a step forward, herein we report on an innovative bioresponsive HA‐based hydrogel (namely, HA‐MMPI) obtained by cross‐linking HA with a branched MMP inhibitor, and on the capacity of HA‐MMPI to inhibit MMP‐2 expression in glioblastoma environment and to reduce tumor cells migration. Due to lack of toxicity and the unprecedented property of modulating the cell microenvironment, HA‐MMPI has promising applications as filler material in the brain cavity residual after surgical resection. Furthermore, as ECM substitute, HA‐MMPI will allow to investigate and characterize the GBM microenvironment during cancer progression, which is essential for new therapies.

## 2 Synthesis of the Branched MMP Inhibitor 1

**Scheme 1:**
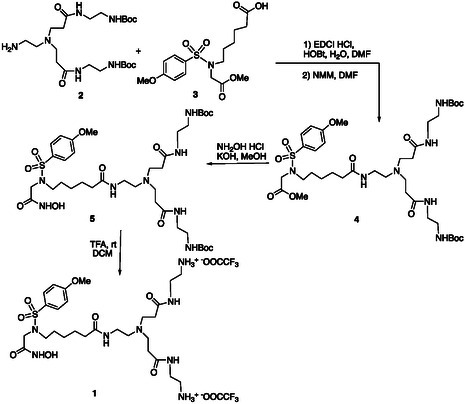
Synthesis of MMP inhibitor **1**.

Upon activation of the carboxylic residue (EDCI, HOBt), the solfonamide derivative **3**, obtained following a reported procedure,^[^ ^27^ ^]^ was reacted with the amino derivative **2** ^[^ ^28^ ^]^ to form derivative **4** (97%). The methyl ester group of **4** was then transformed into the corresponding hydroxamic acid by reaction with hydroxyl amine, affording derivative **5** (50%). Treatment of compound **5** with trifluoroacetic acid gave the branched inhibitor **1** as trifluoroacetate salt (quant.) (**Scheme** **1**).

### 2.1 Synthesis and Characterization of HA‐ MMPI Hydrogel

**Figure 1:**
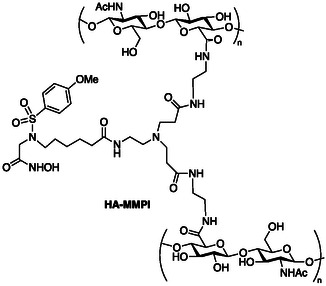
Schematic structure of cross‐linked HA‐MMPI.

In this conceptually original strategy, the branched MMP inhibitor **1** (MMPI) was used to cross‐link HA. Condensation, involving the two free amino groups of MMPI as cross‐linking agents, and the carboxylic groups of the HA chains, was performed in a MMPI/HA molar ratio of 3:1, using EDC and NHS as coupling agents in a 10:1 molar ratio with respect to HA. The generation HA‐MMPI (**Figure** **1**) was confirmed by thermogravimetric (TGA), rheological analysis, Attenatued Total Reflection Fourier Transform Infrared Spectroscopy (ATR‐FTIR), Time of Flight‐Secondary Ion Mass Spectrometry (ToF‐SIMS), and swelling (see Supporting Information for details).

As reported in Figure S2, Supporting Information, in the FTIR spectrum of HA‐MMPI characteristic bands corresponding to HA and the MMPI linker are well detectable. A broad absorption band is observed at ≈3400 cm^−^^1^, which can be attributed to O—H stretching vibrations from the hydroxyl groups of HA and N—H stretching vibrations from amine groups in MMPI. In the region of ≈2920–2850 cm^−1^, C—H stretching vibrations of aliphatic CH_2_ and CH_3_ groups are detectable. The broadening of the Amide I band at ≈1650 cm^−^^1^, due to the C=O stretching of amidic group, supports the formation of amidic bonds between HA and MMPI. This band in fact contains the contribution of the amidic bond formed between the MMP inhibitor and the HA because of cross‐linking reaction, superimposed to those of Amide I C=O vibrations of HA and MMP. Moreover, the ≈1550 cm^−1^ band, due to the amide II vibration, contains the contributions of NH vibration of amidic groups formed by the covalent bonds between HA and MMPI together with those of MMPI. A peak at ≈1400 cm^−1^ is associated with symmetric COO^−^ stretching vibrations from the carboxylate groups in HA. Additionally, the region between ≈1150 and 1050 cm^−1^ shows C—O—C and C—O stretching vibrations, characteristic of the glycosidic linkages in the backbone of HA. Finally, the complex pattern observed in the ≈900–600 cm^−^^1^ region represents the fingerprint region, dominated by C—H bending, sugar ring deformations, and skeletal vibrations of the HA framework.

**Figure 2:**
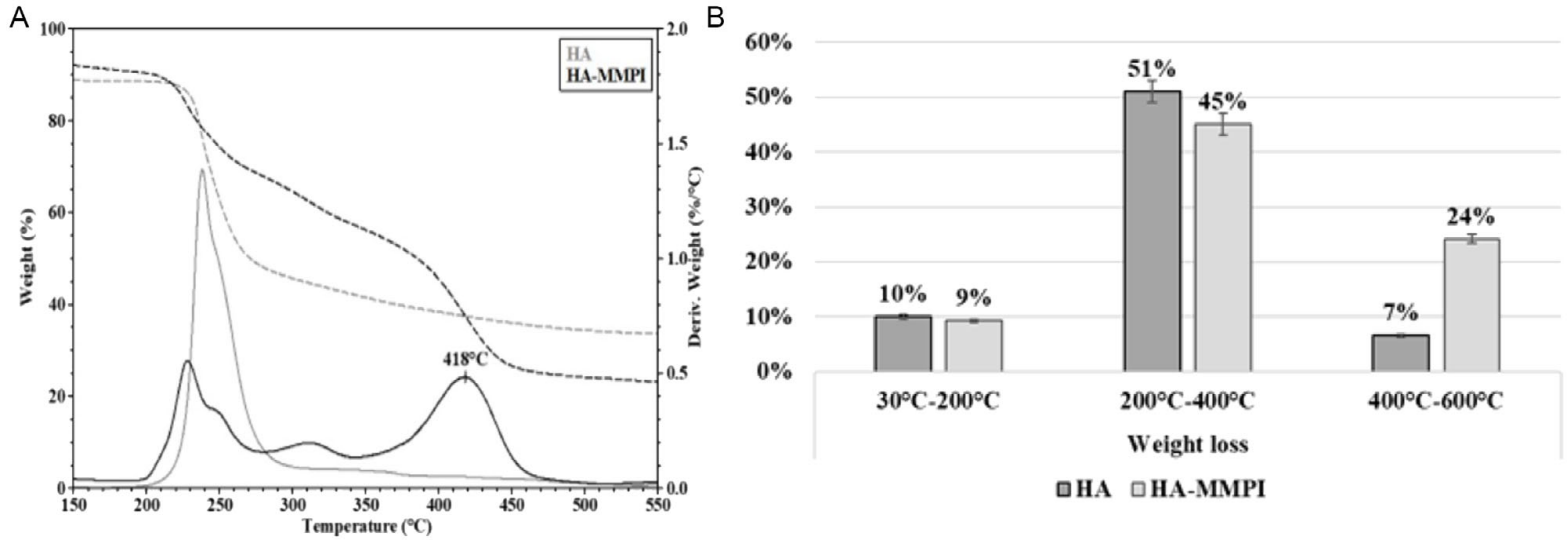
A) TGA analysis of HA and HA‐MMPI. B) Weight loss of HA and HA‐MMPI.

In the ToF‐SIMS analysis of the HA‐MMPI hydrogel are visible distinct mass spectral features confirming the successful cross‐linking of HA with MMPI. In the high *m*/*z* region (≈370–410), both NaHA and HA‐MMPI show signals near *m*/*z* 374–382, characteristic of HA‐derived ions. However, the HA‐MMPI spectrum displays prominent new peaks at *m*/*z* 381 and 403, assigned to the protonated MMPI fragment ([MMPI]^+^) and its carbonyl adduct ([MMPI + C = O]^+^), respectively. In the low *m*/*z* region (≈100–150), NaHA shows typical saccharidic fragments at *m*/*z* 105, 111, 113, and 119, the HA‐MMPI spectrum shows intense peaks at *m*/*z* 107 and 129, attributed to hydrazide and acyl chain fragments from MMPI. The presence of these characteristic MMPI‐derived signals in the HA‐MMPI sample provides evidence of the successful covalent bonding of the MMPI inhibitor in the HA backbone, supporting the molecular design and cross‐linking strategy. The polymeric 3D materials were also characterized based on their response to temperature, by analyzing their weight loss in three temperature ranges (**Figure** **2**).

The total water content of HA‐MMPI (that is the sum of freezable and nonfreezable water) was quantified by TGA, whereas the freezable water was determined by DSC, and by difference, the nonfreezable water was quantified. The weight loss observed in the range from 30 to 200 °C is due to the bulk and hydration water; in the range from 200 to 400 °C the weight loss is related to the degradation of free aliphatic carbon chains, whereas from 400 to 600 °C the degradation of the condensed chains (carbonation) occurs. As summarized in Figure 2, no significant difference was evident between HA and HA‐MMPI in terms of bulk and hydration water. Conversely, HA and HA‐MMPI showed different behaviors for temperature higher than 400 °C. Indeed, above 400 °C HA‐MMPI showed a weight loss larger than HA, as expected for a cross‐linked polymer. The *R*‐value, which is the ratio of the weight loss in the third range of temperature with respect to the second, allows an evaluation of the degree of structuration of the material, in which the higher the *R*‐value, the higher the hydrogel structuring and thermal stability.^[^ ^29^ ^]^ HA‐MMPI has an *R*‐value (0.54 ± 0.02) that is almost 4 times the *R*‐value of HA (0.13 ± 0.01), according to the increase in the material structuring due to the cross‐linking reaction. The mesh size and cross‐linking degree were obtained by rheological analysis. The mechanical properties of HA‐MMPI were evaluated in shear and compression mode by frequency sweep tests and expressed in terms of shear (*G*′ and *G*″) and compression (*E*′ and *E*″) moduli (see Figure S4, Supporting Information). The mechanical properties of HA‐MMPI, evaluated both in shear and compression mode by frequency sweep tests, confirmed the chemical nature of HA‐MMPI hydrogel, being *G*′ > *G*″ and *E*′ > *E*″ in the whole range of frequency. Moreover, being the tan δ value lower than 0.1 in the whole range of frequency, it is also possible to assess that HA‐MMPI is a strong gel in the shear regime (Figure S4, Supporting Information). HA‐MMPI chemical properties, cross‐linking degree, and mesoporosity strictly affect the swelling capability of a hydrogel. HA‐MMPI reached its swelling equilibrium of 99.2% ± 0.5% within 30 min. For HA‐MMPI, the 83% of adsorbed water can be classified as bulk or freezable water whereas the remaining 17% as bound or nonfreezable water. The swelling analysis reported in Figure S5, Supporting Information, showed that cross‐linked HA‐MMPI sample exhibits rapid and finely controlled swelling behavior ideally suited for postresection treatment of GBM. The water content rises by 100% in 10 min. This rapidity is optimal for hydrogels that can easily adhere to the resection cavity. The overall water uptake reaches 60% of maximum in that same period. This swift yet self‐limiting expansion is advantageous to control in a desirable way the interaction with brain tissue and to maintain the inhibitory function of the MMP. Considered together the swelling profile and the rheological properties, the HA‐MMPI features make the hydrogel applicable for the interaction of brain tissue, avoiding the induction of excessive mechanical stress on the surrounding tissue.^[^ ^30^, ^31^ ^]^

### 2.2 Biological Evaluation

**Figure 3:**
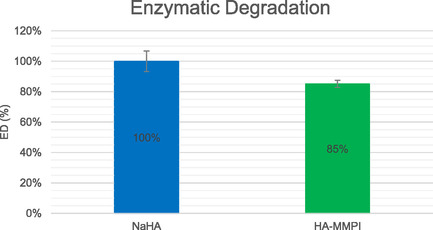
In vitro enzymatic degradation (%) of native HA, NaHA (blue) and cross‐linked HA (HA‐MMPI hydrogel, green) after 3 h of direct contact with hyaluronidase.

First, lack of toxicity of HA‐MMPI was assessed under standard ISO 10 993‐5 (SI Figure S6, Supporting Information). Because one of the main disadvantages in using HA‐based hydrogels is the short life in vivo, due to the presence of hyaluronidases, enzymatic degradation kinetics of HA‐MMPI was then evaluated. Results reported in **Figure** **3** showed an improved stability of cross‐linked HA‐MMPI hydrogel in enzymatic degradation in vitro.

The biological activity of the HA‐MMPI hydrogel was further evaluated by employing 3D U87 GBM spheroids. 3D spheroids models allowed the study of the ability of HA‐MMPI to limit cell detachment and migration from 3D aggregates. The U87 spheroids, which were previously obtained using low attachment plates, were embedded in the HA‐MMPI hydrogels, and in the untreated HA as control, to evaluate both cells migration and the expression of MMP‐2 and of MMP‐9. As a matter of fact, MMP‐2 and ‐9 are both overexpressed in GBM and contribute to the degradation of type IV collagen, which is the most abundant component of the basement membrane.

**Figure 4:**
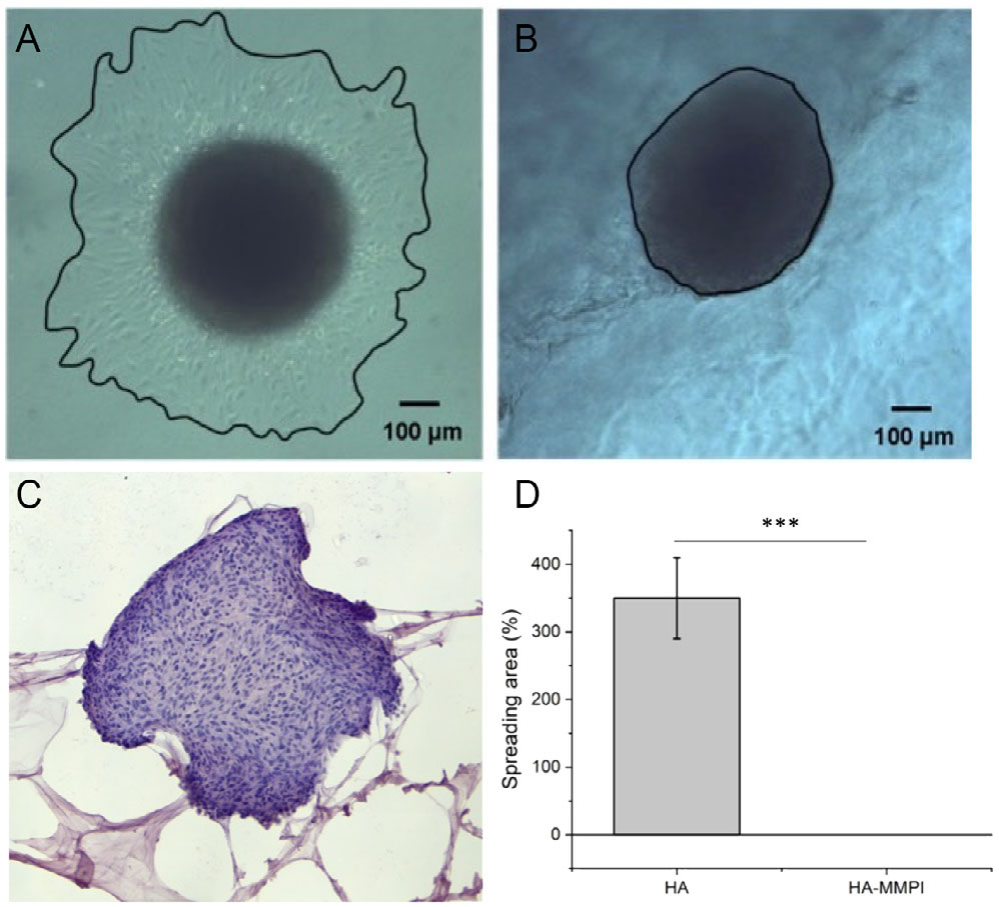
A) Images of U87 spheroid embedded in control HA and B) in functionalized HA‐MMPI‐I. C) Histological images of HA‐MMPI embedded spheroid. D) Spheroid spreading area; data are presented as the mean ± SD, *n* = 11. Statistical significance: *P*‐value < 0.005*, *P*‐value < 0.0025**, and *P*‐value < 0.0005***.

As shown in **Figure** **4**, after 24 h the U87 spheroids embedded in HA began to migrate within the HA matrix (panel A), whereas the spheroids embedded in the HA‐MMPI remained intact (panel B). Considering the pivotal role played by MMPs in invasion and metastasis of cancer cells by proteolysis of ECM components, the ability of HA‐MMPI to maintain compact U87 spheroids has been in‐depth studied by immunohistochemistry (IHC). IHC analysis can indeed provide valuable information to understand the selectivity and effectiveness of the hydrogel system in inhibiting MMP‐2/MMP‐9. The organization of U87 spheroids inside HA‐MMPI hydrogel was thus observed by H&E histological analysis on FFPE spheroids. As shown in Figure 4C, U87 spheroids exhibit a compact structure with no signs of cell migration inside the residual hydrogel. U87 are organized as intertwined bundles, which appear more compact in the external layer and less densely packed in the center. Cells show a spindle morphology with indistinct cytoplasmic borders. They appear well preserved and show moderate anisocytosis and anisokaryosis. These data suggest that the HA‐MMPI hydrogel interacts with the GBM spheroids, having an impact in the ECM remodeling as well.

**Figure 5:**
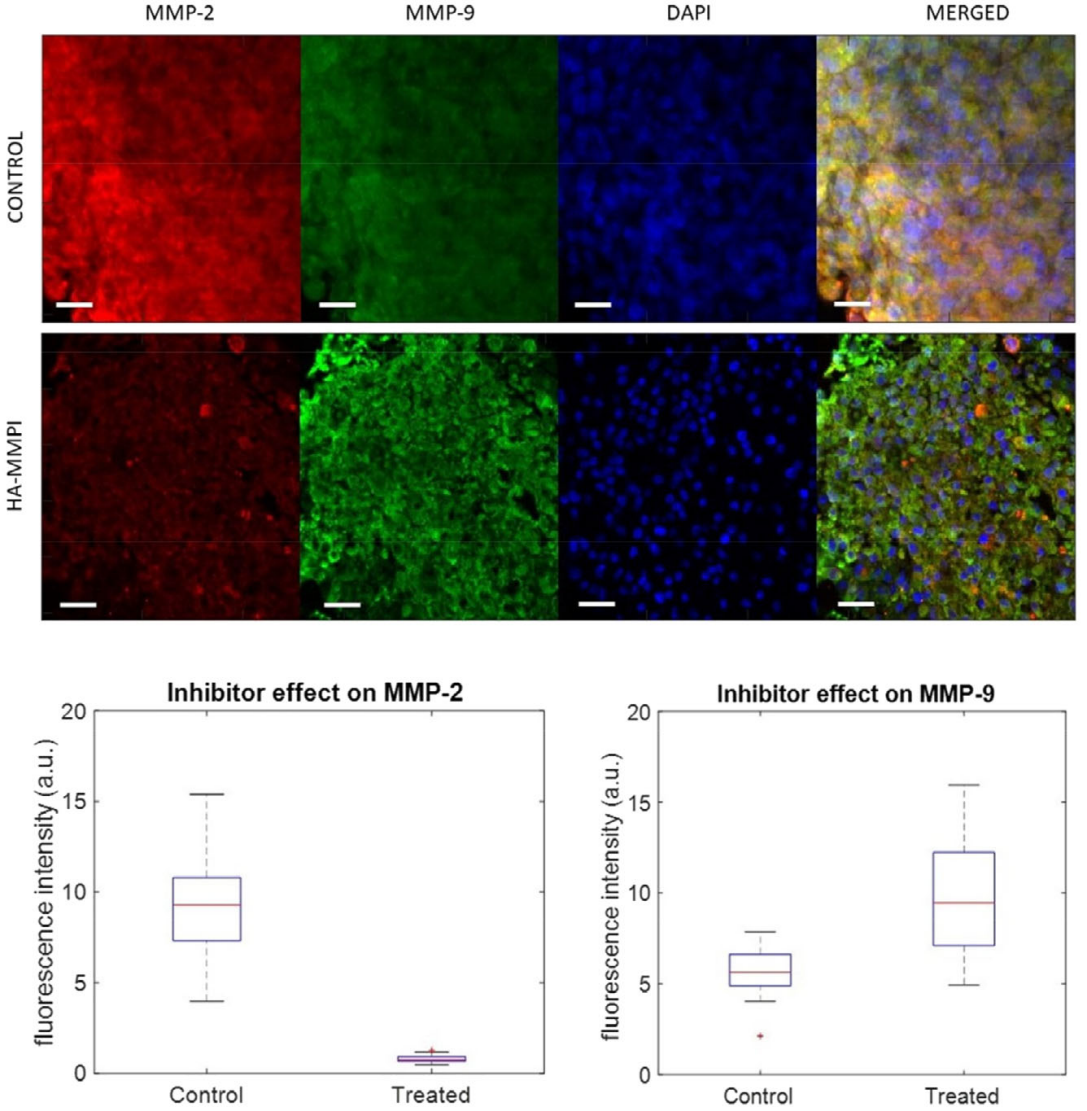
Upper panel: a confocal image of HA‐MMPI and HA (control) spheroids split in the three observed fluorophores CF 488, CF 568, and DAPI as counter‐staining that tag, respectively, MMP‐2, MMP‐9, and nuclei. The fourth image is the result of merged images. Lower panel: box plots of normalized fluorescence intensity channel of MMP‐9 and MMP‐2 for control (HA) and treated (HA‐MMPI) spheroids.

Selectivity of HA‐MMPI toward the two MMPs of interest was evaluated by visualizing and quantifying the localization of both MMPs and by assessing their degree of expression. Using confocal microscopy, the expression of MMP‐2 and MMP‐9 was quantified and compared to the control. **Figure** **5** provides images for the three wavelength channels that are recorded and shows how the CF658 fluorescence significantly decreases, whereas the CF488 fluorescence correspondingly increases. The semiquantitative analysis displayed in Figure 5 (lower panel) supports the qualitative picture observed. Twenty‐four spheroids embedded in HA‐MMPI have been compared to spheroids placed in HA as control. Red fluorescence related to MMP‐2 expression significantly decreased (*P*‐value < 0.001), whereas green fluorescence related to MMP‐9 expression increased (*P*‐value < 0.05). Data are statistically significant, as confirmed in the merged panel, in which overlapped fluorescence is reported. The average fluorescence intensity findings indicate that MMP‐2 expression is downregulated in HA‐MMPI, while MMP‐9 expression is upregulated. In contrast, the control experiment exhibits an increased expression of both MMP‐9 and MMP‐2.^[^ ^32^, ^33^ ^]^ This finding further validates the selectivity of MMPI toward MMP‐2. MMP‐9 is instead a tricky enzyme; in fact, its inhibition might be useful in patients with early‐stage cancers, but it is an anti‐target in patients with advanced disease. Thus, although further investigation is required, MMP‐9 inhibition might be counterproductive in late‐stage cancers.

## 3 Conclusions

In summary, in GBM the extent of resection is associated with survival. However, technical and anatomical issues along with infiltrated tumor cells make a complete tumor resection unpracticable. In this scenario, matrix MMPs negatively impact onto the tumorigenesis of GBM. These enzymes selectively degrade components of the ECM, contributing to cancer cell invasiveness and migratory characteristics by disrupting the basal membrane, even though their expression profile and role remain unclear. In particular, MMP‐2 has an increased activity correlated with the grade of glioma and is considered an attractive target for GBM therapy.^[^ ^34^ ^]^

Herein, we presented a new hydrogel suitable to fill the resection site after GBM surgical resection, and able to inhibit GBM spread. The HA‐based hydrogel we propose is cross‐linked with a branched MMPI; it is nontoxic and more stable to hyaluronidases than the native HA. In GBM microenvironment, this new material can selectively inhibit MMP‐2 in situ. Experiments with U89 spheroids showed that cells migration, responsible for metastasis, is inhibited in HA‐MMPI. Concluding, we believe that the HA‐MMPI hydrogel is a promising tool for the mitigation of tumor relapse and spread.

## Conflict of Interest

The authors declare no conflict of interest.

## Author Contributions

**Federica Barbugian**: data curation (lead); investigation (lead); methodology (lead); writing—original draft (lead). **Domenico Salerno**: data curation (equal); formal analysis (equal); methodology (equal); writing—review and editing (equal). **Elisa Ballarini**: investigation (equal); methodology (equal); writing—review and editing (equal). **Luca Crippa**: investigation (equal); methodology (equal); writing—review and editing (equal). **Oscar** **Francesconi**: funding acquisition (equal); investigation (equal); Supervision (equal); writing—review and editing (equal). **Francesco Mantegazza**: investigation (equal); supervision (equal); writing—review and editing (equal). **Guido Cavaletti**: supervision (equal). **Stefano Roelens**: supervision (equal); writing—review and editing (equal). **Gemma Leone**: investigation (equal); methodology (equal); writing—original draft (equal). **Simone Pepi**: investigation (equal); methodology (equal). **Luigi Talarico**: investigation (equal); methodology (equal). **Agnese Magnani**: conceptualization (equal); supervision (equal); writing—review and editing (equal). **Cristina Nativi**: conceptualization (equal); fund acquisition (lead); supervision (equal); writing—original draft (equal). **Laura Russo**: conceptualization (equal); fund acquisition (lead); project administration (lead); writing—review and editing (equal).

## Supporting information

Supplementary Material

## Data Availability

The data that support the findings of this study are available in the supplementary material of this article.
