# Supplementary material for: Bioresponsive Hyaluronic Acid‐Based Hydrogel Inhibits Matrix Metalloproteinase‐2 in Glioblastoma Microenvironment
Source: ChemMedChem. 2025 Jun 24;20(15):e202401040. doi: 10.1002/cmdc.202401040 (PMC12321271; doi:10.1002/cmdc.202401040)
Supplement: Supplementary file 1 — Supplementary Material [file CMDC-20-e202401040-s001.pdf]

Supporting Information

Bio responsive hyaluronic acid-based hydrogel inhibits MMP-2 in glioblastoma microenvironment

Federica Barbugian, Domenico Salerno, Elisa Ballarini, Luca Crippa, Oscar Francesconi, Francesco Mantegazza, Guido Cavaletti, Stefano Roelens, Gemma Leone, Simone Pepi, Luigi Talarico, Agnese Magnani*, Cristina Nativi*, Laura Russo*

Synthesis of compound 4

A solution of compound 2 (3.50 g, 8.00 mmol) in DMF (30 mL), cooled to 0 °C, was treated with HOBt·H_2O (1.70 g, 12.90 mmol) and EDAC·HCl (2.50 g, 12.90 mmol). The mixture was cooled to 0 °C and stirred. A second solution was prepared by dissolving compound **3** (3.0 g, 6.0 mmol) and NMM (2.0 g, 18.0 mmol) in DMF (20 mL) and cooled to 0 °C. The two solutions were mixed and stirred at rt for 1h, then diluted with CH_2Cl_2. The organic layer was washed with $NaHCO_3$ s.s. (3 x), H_2O (3 x) and brine (1 x) then dried over anhydrous Na_2SO_4. After filtration, the organic solved was removed under vacuum and the crude purified by flash column chromatography on silica gel (CH_2Cl_2/MeOH 9:1) to afford 5.87 g of compound **4**, as brownish glassy solid (97% over two steps).

ESI-MS: *m/z* (%): 844.65 (45) $[M+Na]^+$, 866.83 (100) $[M+Na]^+$ (calculated for $C_{38}H_{65}N_7O_{12}$: 844.04)

^{1}H NMR: (500 MHz, DMSO-d6) δ 7.89 (t, 2H, 2 N*H*), 7.73 (ad, AA' part of a AA'MM'system, J_{AM} = 8.9 Hz, 2H, 2C*H*), 7.6 (t, 1H, N*H*), 7.10 (ad, MM' part of a AA'MM'system, J_{MA} = 8.9 Hz, 2H, 2 C*H*), 6.76 (t, 2H, 2 N*H*), 4.01 (s, 2H, CH_2), 3.84 (s, 3H, CH_3), 3.6 (s, 3H, CH_3), 3.07 (dt, 8H, 4 CH_2), 2.96 (dd, 4H, 2 CH_2), 2.64 (t, 4H, 2 CH_2), 2.41 (t, 2H, CH_2), 2.18 (t, 4H, 2 CH_2), 2.01 (t, 2H, CH_2), 1.42 (m, 4H, 2 CH_2), 1.37 (s, 18H, 6 CH_3), 1.17 (m, 2H, CH_2).

^{13}C NMR: (125 MHz, DMSO -d6) δ 172.3, 172.0, 170.0, 162.9, 156.1, 131.3, 129.6, 114.8, 78.1, 56.1, 52.6, 52.3, 50.0, 48.8, 48.6, 39.1, 37.3, 35.8, 33.7, 28.7, 27.8, 26.1, 25.3.

Synthesis of compound 5

A suspension of $NH_2OH{\cdot}HCl$ (820 mg, 12.0 mmol) in MeOH (5 mL) and a suspension of KOH (985 mg, 18 mmol) in MeOH (2.5 mL) were heated to reflux and the two solutions obtained mixed. The resulting mixture was treated with a solution of **4** (5.00 g, 5.85 mmol) in MeOH (15 mL) and stirred at rt for 30 min. The reaction mixture was treated with HCl (1M, MeOH) until neutralization. After removal of the organic solvent under vacuum, the crude was purified by flash column chromatography on silica gel (CH_2Cl_2/MeOH 9:1) to afford **5** (2.33 g 50%) as a yellow oil.

ESI-MS *m/z* (%): 845.83 (35) $[M+H]^+$, 867.75 (100) $[M+Na]^+$, 844.00 (100) $[M-H]^-$ (calculated for $C_{37}H_{64}N_8O_{12}S$: 845.02).

^{1}H NMR: (500 MHz, DMSO-d6) δ 10.56 (bs, 1H, O*H*), 8.91 (bs, 1H, N*H*), 7.90 (t, 2H, 2 N*H*), 7.77 (ad, AA' part of a AA'MM' system, J_{AM} = 8.8 Hz, 2H, 2C*H*), 7.61 (t, 1H, N*H*), 7.09 (ad, MM' part of a AA'MM' system, J_{MA} = 8.8 Hz, 2H, 2 C*H*), 6.76 (t, 2H, 2 N*H*), 3.85 (s, 3H, CH_3), 3.65 (s, 2H, CH_2), 3.05 (m, 10H, 5 CH_2), 2.96 (dd, 4H, 2 CH_2), 2.64 (t, 4H, 2 CH_2), 2.41 (t, 2H, CH_2), 2.18 (t, 4H, 2 CH_2), 2.02 (t, 2H, CH_2), 1.42 (m, 4H, 2 CH_2), 1.37 (s, 18H, 6 CH_3), 1.15 (m, 2H, CH_2).

^{13}C NMR: (125 MHz, DMSO-d6) δ 172.3, 172.0,160.0, 156.1, 130, 114.7, 78.1, 57, 56.1, 52.6, 52.3, 51.9, 50.0, 49.1, 48.8, 39.1, 37.3, 35.8, 33.7, 28.7, 27.6, 26.2, 25.3.

Synthesis of the bifunctional inhibitor 1

A solution of **7** (2.0 g, 2.0 mmol) in CH_2Cl_2 (25 mL) was treated with TFA (5.50 g, 47.60 mmol) and stirred at rt for 25 min. The organic solvent was then removed under vacuum to give 3.1 g of **1** as trifluroacetate salt (yellow oil, quant.).

ESI-MS *m/z* (%): 645.67 (100) $[M+H]^+$, 643.92 (100) $[M-H]^-$ (calculated for: $C_{31}H_{50}F_6N_8O_{12}S$: 872.84)

^{1}**H NMR** (500 MHz, DMSO-d6) δ 10.66 (bs, 1H, O*H*), 9.52 (bs, 1H, N*H*-9), 8.39 (t, *J* = 5.4 Hz, 2H, 2 N*H*), 8.15 (m, 1H, N*H*), 7.75 (ad, AA' part of a AA'MM' system, J_{AM} = 8.8 Hz, 2H, 2C*H*), 7.85 (bs, 4H, 2 NH_2), 7.10 (ad, MM' part of a AA'MM' system, J_{MA} = 8.8 Hz, 2H, 2 C*H*), 3.97 (s, 2H, CH_2), 3.85 (s, 3H, CH_3), 3.42-3.37 (m, 6H, 3 CH_2), 3.31 (dt, *J* = 6.4 Hz, 4H, 2 CH_2), 3.16-3.19 (m, 2H, CH_2), 3.04 (t, *J* = 7.3 Hz, 2H, CH_2), 2.88 (dt, *J* = 5.8 Hz, 2H, CH_2), 2.63 (t, *J* = 7.2 Hz, 4H, 2 CH_2), 2.08 (t, *J* = 7.3 Hz, 2H, CH_2), 1.47-1.43 (m, 4H, 2 CH_2), 1.21-1.18 (m, 2H, CH_2).

^{13}**C NMR** (125 MHz, DMSO -d6) δ 174.0 (Cq, CO), 170.4 (Cq, CO), 162.9 (Cq), 158.8 (Cq, CO), 158.6 (Cq, CO), 131.1 (*C*q), 129.7 (*CH*), 114.8 (CH), 56.1 (CH_3), 52.2 (CH_2), 49.6 (CH_2), 49.1 (CH_2), 48.0 (CH_2), 39.0 (CH_2), 37.0 (CH_2), 35.6 (CH_2),34.2 (CH_2), 29.3 (CH_2), 27.7 (CH_2), 26.2 (CH_2), 25.1 (CH_2).

Synthesis of HA-MMPI. To 1 g/L sodium hyaluronan solution in ultrapure water (UPW) were added EDC, NHS and the MMPI solution (10 g/L methanolic solution), molar ratio 1:10:10:0.33, respectively, under vigorous mechanical stirring. The reaction was stopped after 2 hours at 25 °C and the obtained crosslinked system was immersed in absolute ethanol. Once completely shrank, it was re-swollen with water. This process was repeated till no more crosslinker was detected in the washing solution. The product was then freeze-dried.

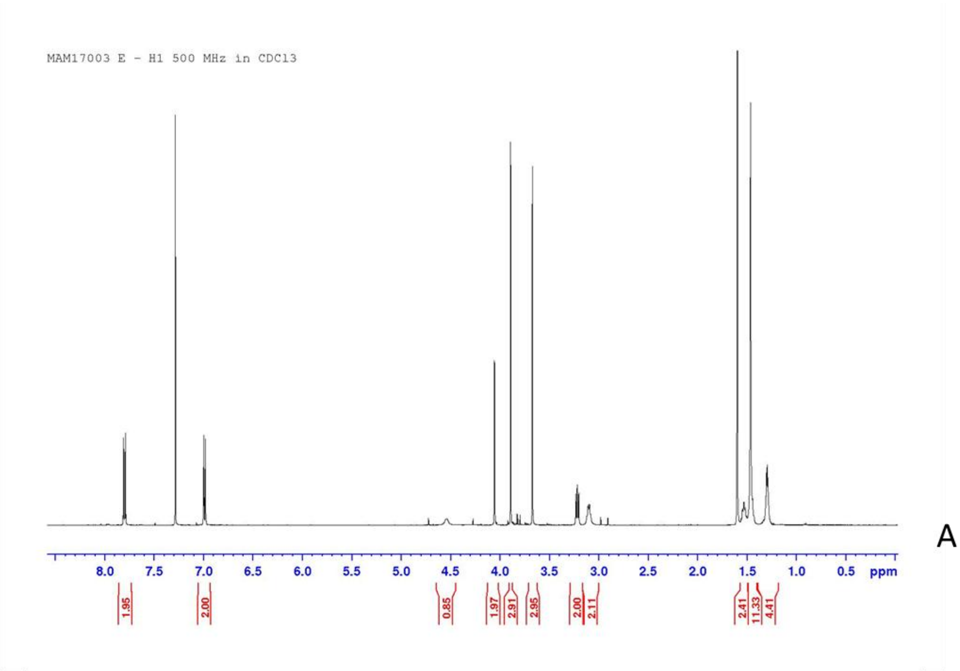
MAM17003 E - H1 500 MHz in CDCl3
8.0 7.5 7.0 6.5 6.0 5.5 5.0 4.5 4.0 3.5 3.0 2.5 2.0 1.5 1.0 0.5 ppm
1.95 2.00 0.85 1.97 2.91 2.95 2.00 2.11 2.41 11.33 4.41

A

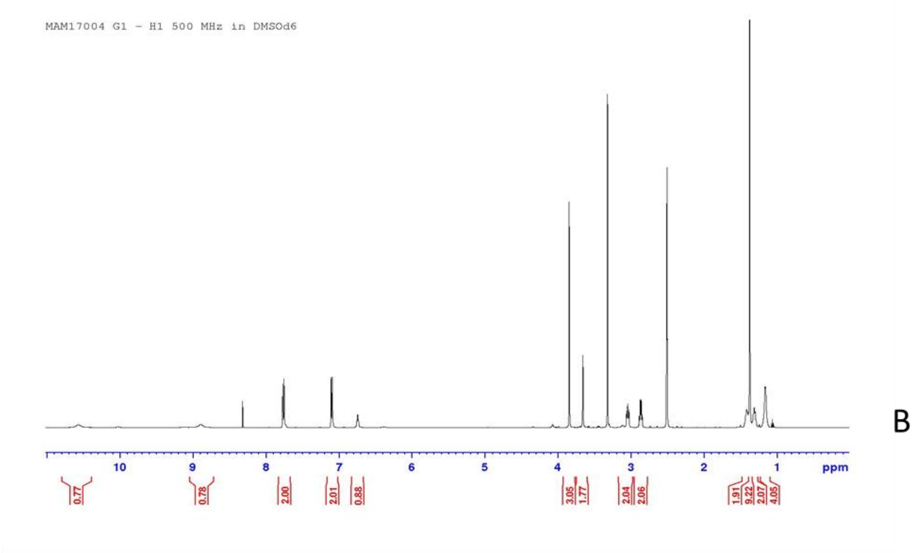
MAM17004 G1 - H1 500 MHz in DMSOd6
10 9 8 7 6 5 4 3 2 1 ppm
0.77 0.78 2.00 2.01 0.88 3.05 1.77 2.04 2.06 1.91 9.22 2.07 4.05

B

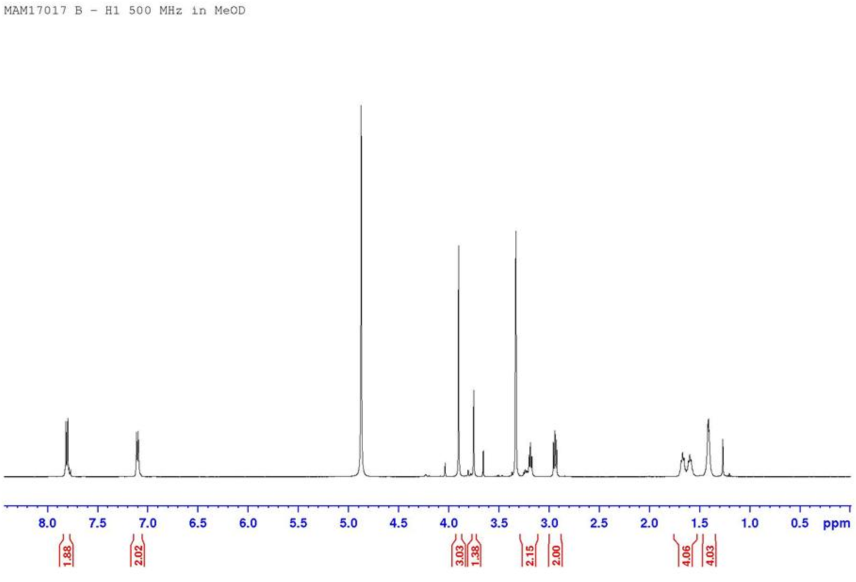
MAM17017 B - H1 500 MHz in MeOD
8.0 7.5 7.0 6.5 6.0 5.5 5.0 4.5 4.0 3.5 3.0 2.5 2.0 1.5 1.0 0.5 ppm
1.88 2.02 3.03 1.38 2.15 2.00 4.06 4.03

C

Figure S1. NMR spectra of: A) N-Boc protected solfonamide methyl ester (compound **4**), B) N-Boc protected hydroxamic acid (compound **5**) and C) MMP inhibitor **1**

Materials and methods

HA-MMPI. Hyaluronan (HA, MW = 2.10MDa) was purchased by JUK Kraeber & co. GMBH. 1-Ethyl-3-3 (dimethylaminopropyl) carbodiimide hydrochloride (EDC) and N-hydroxy-succinimide (NHS) were purchased by Merck KGaA. All the reagents and solvents used for cell culture were purchased by Sigma Aldrich (Germany). Buffer solution pH 6.0, Citric acid/sodium hydroxide solution was purchased from Honeywell Fluka. All other reagents were purchased from Merck KGa, unless differently stated, and used as received without further purifications.

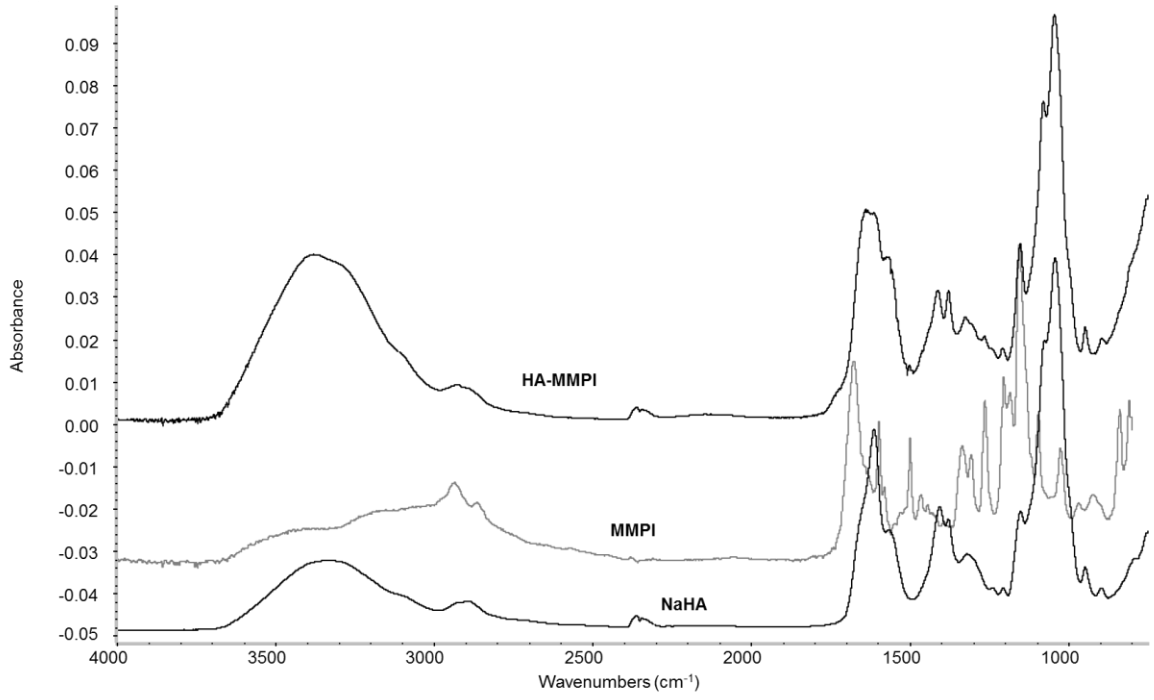

Figure S2. ATR-FTIR spectra of Na HA, HA-MMPI and MMPI.

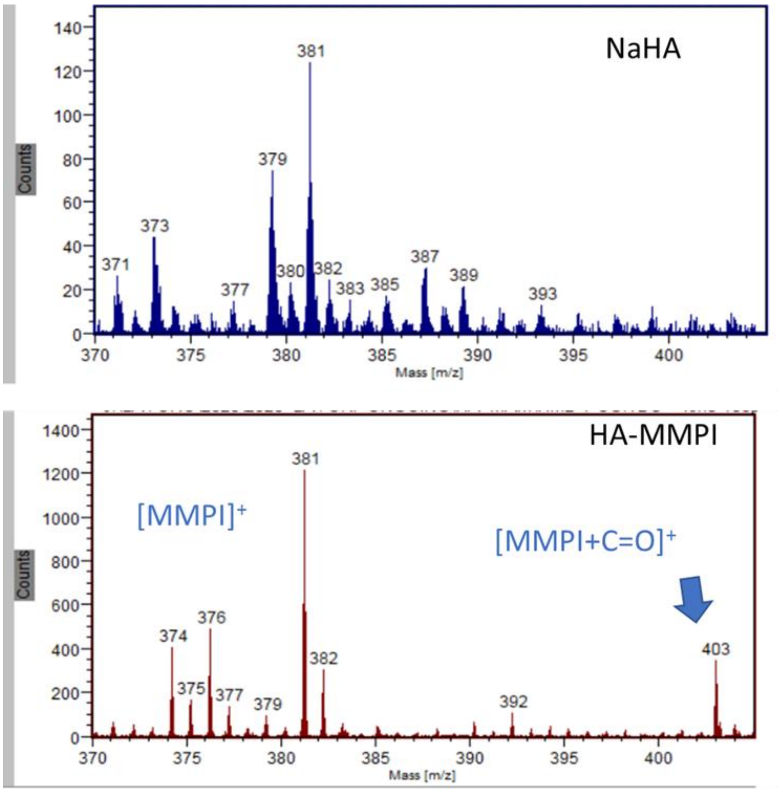
NaHA
Counts
Mass [m/z]
371
373
377
379
380
381
382
383
385
387
389
393
HA-MMPI
[MMPI]+
[MMPI+C=O]+
374
375
376
377
379
381
382
392
403

A

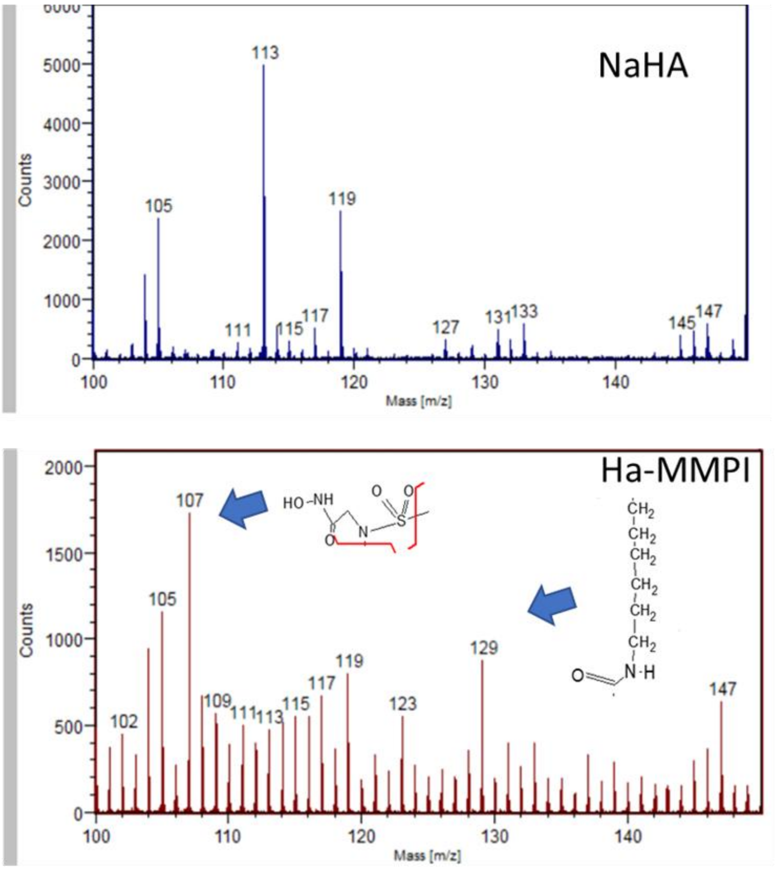
NaHA
Counts
Mass [m/z]
105
111
113
115
117
119
127
131
133
145
147
Ha-MMPI
102
105
107
109
111
113
115
117
119
123
129
147
HO–NH
O
N
S
CH2
N·H

B

Figure S3: A) Characteristic regions (370-425) of positive ion Tof-SIMS spectra of NaHA and HA-MMPI; B) Characteristic regions (100-150) of positive ion Tof-SIMS spectra of NaHA and HA-MMPI

Thermogravimetric Analysis. 10 mg of each sample at dry state were put in a Pt crucible and heated from 30 °C to 900 °C under N_2 (100 mL/min), with a heating rate (HR) of 10 °C/min using SDT-Q600 (TA Instruments)[25].

The mesh size (distance between two crosslinking sites in a hydrogel) of HA-MMPI was calculated from G' value (see Equation 1, SI) and resulted in 17.1 nm ± 0.3 nm. The mesh size results were confirmed by DSC analysis that permitted to calculate the mesoporosity of the hydrogel. Starting from the temperature of water crystallization inside HA-MMPI (– 6.2 °C) and using Equation 2, the mean mesopore size of the material resulted in 11.0 nm ± 0.1 nm. The crosslinking degree, obtained by combining the mesh size result, mesoporosity, and length of the monomer (i.e. 1 nm), resulted 6 % mol/mol.

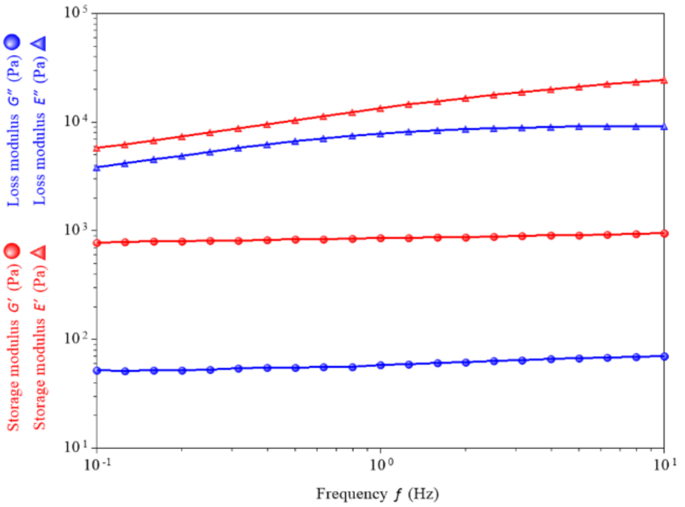

Figure S4. Shear (G' and G'') and compression (E' and E'') mode by frequency sweep tests of HA-MMPI hydrogel

Swelling Analysis. HA-MMPI sample at dry state was immersed in UPW and kept at 37 °C. The hydrogel was weighted every 10 minutes till constant weight, and then to 150 and 240 minutes.

The Water Content was calculated by the Eq. 3:

$$\text{Eq.3} \quad wc = \left(\frac{w_s - w_d}{w_s}\right) \times 100$$

wc, ws and wd are respectively the Water Content, the swollen and the dried weight of the hydrogel.

Types of water: 10 mg of full water swollen sample in UPW was put in a Pt crucible and heated from 30 °C to 300 °C under N2 flow (100 mL/min), HR 10 °C/min using SDT-Q600 (TA Instruments), to obtain the total water .

The weight of the total H_2O content (WH) in the hydrogel sample is expressed in Eq.4:

$$\text{Eq.4} \quad W_H = W_{fH} + W_{nfH}$$

Where W_{fH} is the weight of the freezable H_2O and W_{nfH} is the weight of the non-freezable H_2O.

5 mg of full swollen sample in UPW were sealed in an alodined Al hermetic pan, cooled from room temperature to – 40 °C, (HR 0.2 °C/min.), kept isothermally for 5 minutes and then heated to 40 °C, at HR of 0.2 °C/min. under N2 flow (50 mL/min) using a DS calorimeter Q2000 (TA Instruments), to obtain the freezable water. The freezable H_2O is quantified integrating the endothermic melting peak of the frozen H_2O in the hydrogel sample (ΔHWm) that is then related to the latent heat of melting of the H_2O (ΔHW). The ratio ΔHWm/ΔHW corresponds to the weight of freezable H_2O per gram of full swollen hydrogel (WSG), Eq. 5:

$$\text{Eq. 5} \qquad \frac{W_{fH}}{W_{SG}} = \frac{\Delta H_{Wm}}{\Delta H_W}$$

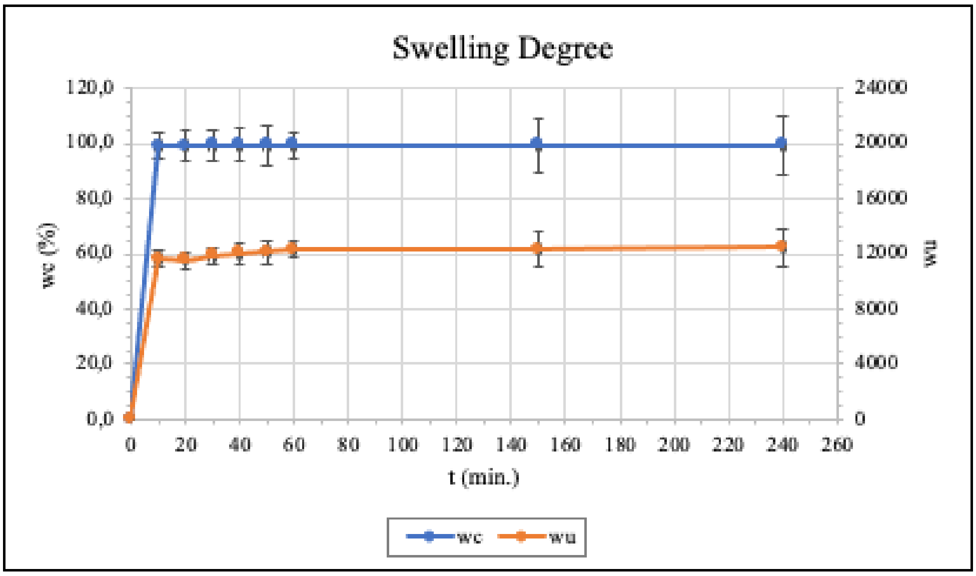

Figure S5. Swelling behavior of HA-MMPI

Rheological analysis. Shear and compression analyses on fully swollen HA-MMPI hydrogel were performed using a Discovery Hybrid Rheometer-2 (DHR-2) (TA Instruments). A plate-plate stainless steel geometry (40 mm upper plate) equipped with Peltier steel plate environmental system was used. The shear and compression frequency sweep test were performed at 37 °C, with a frequency ramp from 0.1 Hz to 10 Hz at a constant strain of 1 %[26]. The mesh size (ξ) was calculated based on the Rubber Elasticity Theory (RET), which correlates the mean dimension of the mesh with G', (Eq. 1)[27] :

$$\xi = \left(\frac{G' N_A}{R\,T}\right)^{-\frac{1}{3}} \quad \text{(Eq 1)}$$

Where G' is the elastic modulus in Pa, NA is the Avogadro number, R is the universal gas constant in J·K-1·mol-1, T is the temperature in K.

Mesopore size analysis. 5 mg of full water swollen sample in UPW were sealed in an alodined Al hermetic pan, cooled from room temperature to – 60 °C, (at HR of 5 °C/min), then heated from – 60 °C to – 0.3 °C at 5 °C/min., and kept isothermally for 10 minutes. Finally, it was cooled down again at – 60 °C/min. with a heating rate of 0.2 °C/min. All steps were performed under N2 flow (50 mL/min) using a DS calorimeter Q2000 (TA Instruments). The mean mesopore radius was then calculated from the Eq2 [28]:

$$Rp\ (nm) = \left(\frac{-64.67}{\Delta T}\right) + 0.57 \quad \text{(Eq. 2)}$$

Where ΔT is the difference from the temperature of the peak of freezing of H2O in the hydrogel and the triple point of H_2O (Tp = - 0.01 °C)

In vitro cytotoxicity and cytocompatibility: cell viability

The cytotoxicity of the samples was evaluated using the direct contact test as reported in ISO 10993-5. The cytotoxicity of HA-MMPI towards NIH3T3 was assessed and the results of the test are reported in Figure S6

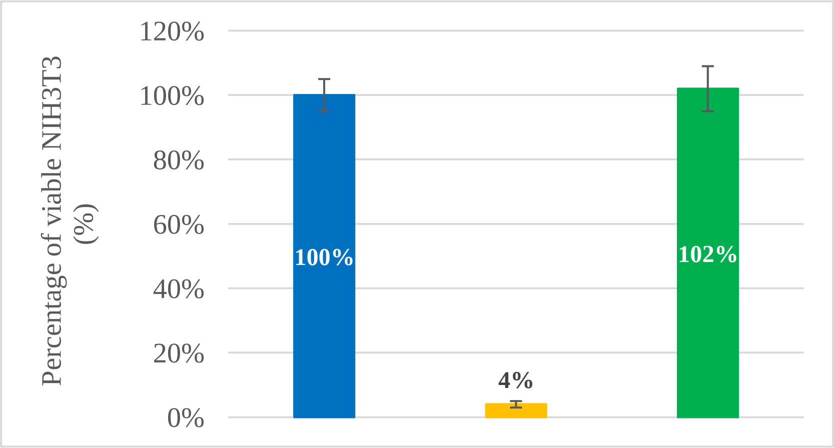

Figure S6. Direct contact test: percentage of viable NIH3T3 after 24 hours of contact: negative control (complete medium) (blu); positive control (PVC) (orange); HA-MMPI hydrogel (green)

The crosslinked polysaccharide does not exert any negative effects on NIH3T3 cell proliferation: it has a superimposable effect with the negative control, so it is not cytotoxic.

Cell culture and 3D Spheroids formation. U87 human glioblastoma cell line was obtained from Sigma-Aldrich (Milan, Italy). U87 MG was grown in Minimum Essential Medium (MEM) supplemented with 2 mM L-glutamine, 1.0 mM sodium pyruvate, 10% fetal bovine serum, 100 units/ml penicillin, and 100 mg/ml streptomycin. Cell culture was maintained in 2D monolayers at 37 ° C under a humidified atmosphere with 5% CO_2. Cell aggregation was induced by growing cell suspensions in 96-multiwell Ultra-low Attachment Surface as already described. Briefly, once cell culture monolayers were detached via standard trypsinization, cells were counted and resuspended to reach the concentration of 5×10^4/ml. 100 µl of cell suspension (5×10^3) were pipetted into each well and allowed to grow for 5 days until spheroids reach the diameter of ~500 µm.

Spheroid cultures in HA-MMPI. 15 mg of HA-MMPI has been hydrated with 1 ml of PBS at pH 7,4. Once the buffer has been completely absorbed, the sample was split in 3 and inserted into a 96 well plate. In the meantime, after 5 days of growth, 10 resulting spheroids (diameter ~500 µm) were moved by 50 µl gentle pipetting fresh culture medium into PBS hydrated HA-MMPI coated well plate and cultured for 1 day.

Immunofluorescence and Histological Analysis. Embedded spheroids were fixed with 10% neutral buffered formalin for 2 h at RT, washed with phosphate-buffered saline (PBS), placed between 2 pieces of filter paper, and moved into histological cassettes. Using a standard protocol, samples were paraffin-embedded with a tissue processor (ETP, Histo-Line Laboratories) and cross-sectioned by rotatory microtome (Leica RM2265). Sections at 3 µm thickness were obtained, mounted on glass slides, and stained with Haematoxylin and Eosin (H&E). Representative images were captured with a light microscope (Olympus BX51) equipped with a digital camera (Evolution VF digital Camera) using Image-Pro Plus software. Embedded spheroids were employed for immunofluorescence assays for MMP-2 and MMP-9 detection. Samples were deparaffinized with

xylene and rehydrated through a 100%, 95%, 70%, and 50% ethanol series and lastly washed in water. Heat induced antigen retrieval was performed using a Citrate buffer solution pH 6 at 90°C for 10 min. After cooling for 20 min at RT, slices were incubated in blocking solution (5% goat serum, 1% Triton X-100 in PBS) at room temperature for 30 min. The sample was incubated overnight at 4°C with a primary antibody against anti-MMP-9 (anti-MMP-9 produced in mice, SAB1402274) and MMP-2 (anti-MMP-2 produced in rabbit, SAB2108458) diluted 1:100 in PBS 1% goat serum. After incubation, slices were washed 3 times for 5 min with 1:5 diluted blocking solution. The secondary antibodies used were CF 488-conjugated goat anti-mouse IgG (SAB46000234) and CF 568-conjugated goat anti-rabbit IgG (SAB4600082) 1:200 diluted in PBS for 1 h at RT. After 3 PBS washes, 5 min each, coverslips were mounted with a drop of Fluoroshield™ Mounting Medium with DAPI to visualize the nuclei and imaged by confocal fluorescence microscopy. As a control, the same staining protocol has been followed on spheroids without the HA-MMPI biomaterial.

Computation of the Area reduction. We claim that estimating the effect of the treatment on the spheroid is more efficient when evaluating the reduction of the spheroid's area rather than the reduction of the spheroid's diameter. This is due to the fact that a slight reduction of the diameter implies the annihilation of a circular area close to the border of the spheroid which includes many cells. Therefore, we would risk to underestimate the effect of the treatment when detecting what appears to be a minor reduction of the diameter instead of considering how many points of the spheroid, thus how much area, were suppressed by the treatment. In mathematical terms, we approximate the area of the spheroid's section as the area of a circle. Before any treatment, we assume this area to be:

$$A_i = \pi\left(\frac{d_i}{2}\right)^2,$$

where d_i is the diameter of the spheroid. After the treatment, we apply again the formula above after replacing d_i with d_f, being the diameter of the spheroid after the treatment.

By taking the percentage of the relative difference between the areas, we get that the treatment reduced the area of the spheroid by a factor of 32.7%.

Confocal Analysis. Confocal images were acquired with a Zeiss cell observer spinning disk microscope equipped with a sCmos camera (Hamamatsu Orca Flash V2.0) using a 10X magnification objective. Three solid-state lasers with the following wavelengths 405, 488, and 561 nm provide the fluorescence excitation, and a single multiband fluorescence cube with the following spectra windows 459/20 525/30 610/40 was used to filter the fluorescence signal. For each sample were acquired multiple z-stack images with a lateral dimension of 350x350 μm and equispaced of 1.5 μm in the z-direction.

A Matlab ad-hoc code was developed for the semi-quantitative analysis of the fluorescence intensity. The first operation was to select for each section the image with the highest global fluorescence in the acquired z stack. Following the image is automatically masked by fixing an intensity threshold that excludes the image region where are not present cells. Finally, the spatial average fluorescence intensity of the masked image is calculated for both the green and red channels.
